# Nutrient digestibility, characteristics of rumen fermentation, and microbial protein synthesis from Pesisir cattle diet containing non-fiber carbohydrate to rumen degradable protein ratio and sulfur supplement

**DOI:** 10.14202/vetworld.2024.672-681

**Published:** 2024-03-22

**Authors:** Mardiati Zain, Ujang Hidayat Tanuwiria, Jasmal Ahmari Syamsu, Yunilas Yunilas, Roni Pazla, Ezi Masdia Putri, Malik Makmur, Ummi Amanah, Putri Okta Shafura, Bima Bagaskara

**Affiliations:** 1 Department of Animal Nutrition and Feed Technology, Faculty of Animal Science Andalas University, Kampus Limau Manis, Padang, West Sumatera, Indonesia; 2 Ruminant and Feed Chemistry Laboratory, Department of Animal Nutrition and Feed Technology, Faculty of Animal Sciences, Universitas Padjadjaran. Jl. Raya Bandung-Sumedang Km. 21, Jatinangor, Sumedang 45363, West Java, Indonesia; 3 Department of Animal Nutrition, Faculty of Animal Sciences, Universitas Hasanuddin, Jl. Perintis Kemerdekaan KM. 10 Kampus UNHAS Tamalanrea, Makassar; 4 Faculty of Agriculture, Department of Animal Science, Universitas Sumatera Utara, Medan; 5 Research Center for Animal Husbandry, National Research and Innovation Agency (BRIN), Jl. Raya Jakarta Bogor 11, Cibinong 16915, Indonesia

**Keywords:** degradable and undegradable protein, digestibility, non-fiber carbohydrate, rumen fermentation, sulfur

## Abstract

**Background and Aim:**

To achieve optimal feed efficiency in ruminants, especially Pesisir cattle, it is necessary to maintain a harmonious equilibrium between energy and protein levels within the rumen. Sulfur supplementation can potentially escalate the energy–protein balance in the rumen. The aim of this study was to explore the formulation of ruminant diets by synchronizing rumen degradable protein (RDP) and non-fiber carbohydrate (NFC) while adding sulfur minerals at different levels. Nutrient digestibility, NH_3_ concentration, volatile fatty acids (VFA) production, microbial protein synthesis (MPS), and methane gas production were assessed.

**Materials and Methods:**

We employed a randomized block design with a 2 × 2 × 3 factorial arrangement and examined diverse incubation periods of 6, 24, and 48 h. Treatment consisted of RDP (60% and 65%), NFC (35% and 40%), and sulfur (0%, 0.15%, and 0.3%) levels. In this study, the Tilley and Terry *in vitro* technique, which used Pesisir cattle’s rumen fluid, was employed to assess the digestibility of dry matter, organic matter, acid detergent fiber, neutral detergent fiber, and RDP-Rumen undegradable protein. In addition, it measures various rumen fluid attributes, including pH, NH_3_, VFA, MPS, and methane gas production.

**Results:**

Treatment with a coordinated combination of 65% RDP and 40% NFC combined with 0.15% sulfur supplement yielded significantly improved digestibility and notably reduced methane gas production (p < 0.05).

**Conclusion:**

The enhancement in digestibility and reduction in methane gas emissions can be attributed to the interaction of RDP, NFC, and sulfur. Feed digestibility was increased in the 65% RDP treatment with 40% NFC and 0.15% sulfur, along with a decrease in methane gas production.

## Introduction

Feed formulations based on crude protein requirements are considered less effective in meeting the protein needs of ruminant livestock, especially in high-producing livestock. For ruminant livestock, protein needs come from microbial proteins that lyse into the post-rumen and bypass proteins. Rumen microorganisms synthesize proteins by incorporating nitrogen (N) from rumen degradable protein (RDP), which easily breaks down in the rumen. However, the bypass protein is difficult to degrade by rumen microbes, so it bypasses directly to the post-rumen. This is called rumen undegradable protein (RUP) or undegradable rumen protein. The balance between RDP and RUP in the diet optimizes the protein needs of ruminants. The use of RDP in diet requires an appropriate source of easily degradable carbohydrates in the rumen non-fiber carbohydrate (NFC) so that the availability of protein and energy to synthesize rumen microbial protein is synchronized. The function of rumen microbes is to digest food substances for ruminant livestock, and the lysed microbes will become a source of protein for the host. Therefore, increasing the rumen microbial population is essential for increasing feed digestibility and protein availability for the host animal [1]. We found that RDP-NFC-based diet still needs further study to improve its potential for ruminant livestock, especially high-production livestock. Advances in RDP-NFC-based diets can be achieved by supplementing ruminants with minerals that play a crucial role.

Minerals are needed in relatively small quantities but play a very important role in animal feed. Various minerals significantly enhance rumen microbial activity [2]. Sulfur often limits the growth of rumen microbes [3]. To optimize the breakdown of feed within the rumen, it is essential to ensure an adequate supply of minerals. Sulfur is a vital mineral that promotes the development and function of rumen microorganisms. Sulfur is the first limiting nutrient for the efficiency of rumen fermentation and has a major effect on the supply of microbial protein to livestock. This mineral content is very low and is often depleted in feed from tropical regions and in feed from agricultural and plant waste. In addition, the bioavailability of minerals in fiber feed is also low [4]. In fact, we need to supplement our diet with minerals. Sulfur is utilized by rumen microbes to form three sulfur-containing amino acids (methionine, cystine, and cysteine). In addition, sulfur is a source of vitamins thiamin and biotin. Therefore, it is necessary to supplement the diet with sulfur to optimize the fermentation process in the rumen.

Pesisir cattle, a local genetic resource found in the West Sumatra province of Indonesia, have the advantage of adapting to harsh environments, making it an excellent candidate for beef cattle that can thrive in the tropics. Compared with other tropical cattle breeds, Pesisir cattle exhibit reduced cooking loss and higher protein content in their meat [5]. To date, research on the nutritional requirements for the development of Pesisir cattle has not been extensive and has been limited to the identification of potential feed sources and their use [6, 7]. On the basis of the above considerations, more in-depth nutritional studies in the form of synchronizing degradable proteins and soluble carbohydrates with sulfur supplementation may optimize rumen function for sustainable growth of Pesisir cattle. We found a lack of data on sulfur supplementation in RDP-NFC-based diet.

Therefore, this study aimed to evaluate the RDP-NFC-based diet supplemented with sulfur by observing nutrient digestibility, NH_3_ concentration, volatile fatty acids (VFA) production, microbial protein synthesis (MPS), and methane gas production in Pesisir cattle through *in vitro* method.

## Materials and Methods

### Ethical approval

Because no animals were used in this study, there is no need for ethical approval. Rumen fluid was obtained from the slaughtered cattle of a slaughterhouse.

### Study period and location

The study was conducted from June to August 2023 at the Ruminant Nutrition Laboratory of the Faculty of Animal Husbandry at Andalas University.

### Experimental diets

The experimental diets consisted of elephant grass, *Indigofera zollingeriana*, *Gliricidia sepium*, corn, bran, tofu dregs, top mix, and sulfur minerals according to treatment. In this study, a 2 × 2 × 3 treatment factorial design was implemented using a randomized block design, which consisted of two levels of RDP (60% and 65%), two levels of NFC (35% and 40%), and three levels of sulfur (0%, 0.15%, and 0.3%). The experimental diet consisted of 50:50% forage and concentrate (based on dry matter [DM]). Nutrients in the experimental diet are presented in Table-1.

**Table-1:** Chemical composition of experimental diets (%).

Treatments												
RDP	60						65					
NFC	35			40			35			40		
Sulfur	0	0.15	0.3	0	0.15	0.3	0	0.15	0.3	0	0.15	0.3
Feed compositions												
Elephant grass	40	40	40	40	40	40	30	30	30	37	37	37
*Indigofera zollingeriana*	10	10	10	7	7	7	3	3	3	7	7	7
*Gliricidia sepium*	10	10	10	13	13	13	27	27	27	16	16	16
Coconut cake	9	9	9	7	7	7	4	4	4	3	3	3
Corn	16	16	16	5	5	5	20	20	20	12	12	12
Rice bran	12	12	12	14	14	14	4	4	4	3	3	3
Tofu waste	2	2	2	13	13	13	11	11	11	21	21	21
Mineral	1	1	1	1	1	1	1	1	1	1	1	1
Chemical compositions												
CP	15.34	15.34	15.34	15.04	15.04	15.04	16.75	16.75	16.75	16.48	16.48	16.48
TDN	65.09	65.09	65.09	65.02	65.02	65.02	66.23	66.23	66.23	66.46	66.46	66.46
Crude fiber	21.52	21.52	21.52	21.97	21.97	21.97	20.39	20.39	20.39	20.10	20.10	20.10
Dry matter	88.60	88.60	88.60	89.15	89.15	89.15	89.74	89.74	89.74	89.64	89.64	89.64
OM	89.60	89.60	89.60	90.15	90.15	90.15	90.74	90.74	90.74	90.64	90.64	90.64
Ash	10.40	10.40	10.40	9.85	9.85	9.85	9.26	9.26	9.26	9.36	9.36	9.36
EE	4.03	4.03	4.03	3.69	3.69	3.69	3.51	3.51	3.51	3.04	3.04	3.04
NFE	48.71	48.71	48.71	49.46	49.46	49.46	50.09	50.09	50.09	51.01	51.01	51.01
NDF	33.92	33.92	33.92	29.91	29.91	29.91	33.62	33.62	33.62	29.32	29.32	29.32
ADF	20.36	20.36	20.36	18.17	18.17	18.17	18.70	18.70	18.70	17.64	17.64	17.64
CEL*	16.74	16.74	16.74	15.26	15.26	15.26	14.68	14.68	14.68	14.67	14.67	14.67
HEMI*	13.56	13.56	13.56	11.75	11.75	11.75	14.92	14.92	14.92	11.68	11.68	11.68
LIG*	2.90	2.90	2.90	2.23	2.23	2.23	3.49	3.49	3.49	2.34	2.34	2.34
SIL*	0.71	0.71	0.71	0.67	0.67	0.67	0.53	0.53	0.53	0.63	0.63	0.63

RDP=Rumen degradable protein, NFC=Non-fiber carbohydrate, CP=crude protein, TDN=True digestibility Nutrient , OM=organic matter, EE=extract ether, NFE=nitrogen-free extract, ADF=Acid detergent fiber, NDF=Neutral detergent fiber,

* CEL=Cellulose, HEMI=Hemicellulose, LIG=Lignin, SIL=Silica

### *In vitro* procedure and sample measurement

We performed the *in vitro* investigation using the method described by Tilley and Terry [8]. This method is widely used, and Tilley and Terry confirmed a high correlation between *in vitro* and *in vivo* digestibility. Rumen fluid was obtained from Pesisir cattle slaughtered at a slaughterhouse which was fed with forage and concentrate. Rumen fluid was filtered through a nylon mesh with a pore size of 100 μm and bubbled with CO_2_ in a water bath shaker at 39°C. Filtered rumen fluid was diluted 1:4 with a buffer solution (McDonald, 1947). A 2.5-g sample was placed into an Erlenmeyer tube, and 250 mL of rumen fluid and buffer solution were added. *In vitro* feed fermentation in this experiment was performed in an incubator shaker with three types of incubation times: 6 h, 24 h, and 48 h. The fermentation process shall be terminated by immersing the Erlenmeyer tube in a block of ice. Subsequently, the pH of the rumen was measured using a pH meter, and the supernatant was separated from the residue using centrifugation. The rumen fermentation characteristics were evaluated in the supernatant, while the residue was used for feed digestibility analysis. The resulting supernatant was stored in the freezer until NH_3_, volatile fatty acids (VFA), and Microbial Protein Synthesis (MPS) analysis was required. The NH_3_ concentration was computed following the procedure outlined by Conway and O’Malley [9]. Conway and O’Malley techniques are widely used, and it is simple to perform. Partial VFA concentration was quantified by gas chromatography. Methane gas production (CH_4_) was calculated from partial VFA data using the following formula [10]: CH_4_ = 0.45 × acetate–0.275 × propionate + 0.40 × butyrate. The MPS assessment followed the procedure outlined by Lowry *et al*. [11].

### Statistical analysis

Data collected in this study were analyzed using the Statistical Package for the Social Sciences version 25 software (IBM SPSS Statistics, NY, USA). Duncan’s multiple range test was employed to examine the variations among the treatment groups following this analysis. A quadratic regression model using Minitab version 20.3 (Solutions Analytics, Pennsylvania, USA) was applied to process the data for estimating the rumen methane production.

## Results

### Nutrient digestibility

Diet digestibility increased significantly (p < 0.01) with increasing RDP levels. The NFC content and the ratio of RDP, NFC, and sulfur also had a significant (p < 0.05) effect on digestibility. *In vitro* of dry matter digestibility (IVDMD) and *In vitro* of organic matter digestibility (IVOMD) with an incubation time of 48 h increased from 54.27% to 62.77% and 55.72% to 63.89%, respectively. *In vitro* of acid detergent fiber digestibility (IVADFD) varied between 59.20% and 69.83%. There was an increase in *In vitro* of Neutral detergent fiber digestibility (IVNDFD), RDP increased from 56.54% to 66.69%, RUP varied between 68.46% and 80.67%, and RUP ranged from 19.33% to 31.54%. Table-2 lists IVDMD, IVOMD, IVADFD, IVNDFD, RDP, and RUP.

**Table-2:** IVDMD, IVOMD, IVADFD, IVNDFD, RDP, and RUP (%) of experimental diets.

Time	IVDMD											

	111	112	113	121	122	123	211	212	213	221	222	223
6 h	24.38^ab^	24.57^ab^	19.82^bc^	24.90^ab^	28.67^a^	27.60^a^	27.37^a^	27.51^a^	25.42^ab^	20.82^bc^	17.82^c^	17.31^c^
24 h	41.00^c^	40.97^c^	42.26^bc^	53.87^a^	48.68^bc^	51.01^abc^	51.87^ab^	51.64^ab^	51.94^ab^	54.08^a^	55.84^a^	57.30^a^
48 h	55.65^ab^	56.07^ab^	54.27^b^	55.07^ab^	56.65^ab^	56.40^ab^	61.22^ab^	58.90^ab^	60.12^ab^	60.91^ab^	62.77^a^	61.45^ab^

**Time**	**IVOMD**											

	**111**	**112**	**113**	**121**	**122**	**123**	**211**	**212**	**213**	**221**	**222**	**223**

6 h	24.02^bc^	23.82^bc^	19.67^cd^	25.02^ab^	22.56^c^	27.56^a^	27.20^a^	27.84^a^	23.39^c^	18.26^cd^	17.72^d^	17.87^d^
24 h	39.75^e^	46.92^de^	43.71^de^	55.30^ab^	49.90^cd^	51.24^ab^	50.13^ab^	50.83^ab^	52.85^b^	53.79^b^	56.97^ab^	58.66^a^
48 h	57.03^ab^	57.50^ab^	55.72^b^	56.48^ab^	58.17^ab^	58.07^ab^	62.07^ab^	58.68^ab^	57.32^ab^	60.79^ab^	63.89^a^	62.33^ab^

**Time**	**IVADFD**											

	**111**	**112**	**113**	**121**	**122**	**123**	**211**	**212**	**213**	**221**	**222**	**223**

6 h	34.25^bc^	34.31^bc^	33.31^bc^	32.22^b^	34.75^bc^	38.12^ab^	38.93^ab^	42.52^ab^	43.90^a^	43.64^a^	40.99^ab^	24.49^c^
24 h	58.46^ab^	62.83^ab^	60.61^ab^	58.01^ab^	57.70^ab^	54.26^b^	60.96^ab^	60.17^ab^	60.92^ab^	61.68^ab^	62.68^ab^	65.75^a^
48 h	62.21^a^	67.10^bc^	59.20^de^	62.71^a^	57.12^e^	60.31^de^	64.23^cd^	62.55^a^	65.11^abc^	69.79^ab^	69.83^ab^	68.11^bc^

**Time**	**IVNDFD**											

	**111**	**112**	**113**	**121**	**122**	**123**	**211**	**212**	**213**	**221**	**222**	**223**

6 h	29.84^b^	32.48^b^	27.63^b^	34.48^ab^	34.68^ab^	36.02^ab^	32.82^b^	31.16^b^	37.35^a^	28.71^b^	29.97^b^	25.06^c^
24 h	45.93^e^	47.08^e^	46.36^e^	56.28^ab^	49.34^e^f	52.59^de^	51.61^d^	51.84^d^	54.00^cd^	54.89^cd^	57.05^ab^	58.27^ab^
48 h	57.22^cd^	57.38^cd^	56.73^d^	56.54^d^	60.07^cd^	58.06^cd^	64.92^bc^	62.60^bc^	63.73^bc^	66.69^ab^	66.16^ab^	63.11^bc^

**Time**	**RDP**											

	**111**	**112**	**113**	**121**	**122**	**123**	**211**	**212**	**213**	**221**	**222**	**223**

6 h	51.52^e^	53.36^e^f	57.44^d^	56.86^de^	56.70^de^	56.83^de^	66.90^ab^	66.79^ab^	64.84^b^	61.33^c^	66.22^ab^	68.55^a^
24 h	60.55^e^	60.86^e^	62.00^de^	55.58f	64.85^cd^	65.56^c^	62.01^de^	68.91^b^	64.61^cd^	66.61^bc^	73.96^a^	74.57^a^
48 h	68.46^e^	77.17^bc^	73.80^cd^	74.01^d^	76.95^bc^	74.48^d^	79.21^bc^	77.96^bc^	73.83^d^	80.41^ab^	80.67^ab^	72.05^a^

**Time**	**RUP**											

	**111**	**112**	**113**	**121**	**122**	**123**	**211**	**212**	**213**	**221**	**222**	**223**

6 h	48.48^a^	46.65^ab^	42.57^c^	43.18^bc^	43.30^bc^	43.17^bc^	33.10^ef^	33.21^ef^	35.16^e^	38.67^d^	33.79^ef^	31.45^f^
24 h	39.46^b^	39.14^b^	38.01^bc^	44.42^a^	35.15^cd^	34.44^d^	37.99^bc^	31.10^e^	35.39^cd^	33.40^de^	26.04^f^	25.43^f^
48 h	31.54^a^	22.84^bc^	26.20^b^	25.99^b^	23.05^bc^	25.52^b^	20.79^cd^	22.04^cd^	26.17^b^	19.59^cd^	19.33^cd^	27.95^b^

111=RDP 60%, NFC 35%, Sulfur 0%; 112=RDP 60%, NFC 35%, Sulfur 0.15%; 113=RDP 60%, NFC 35%, Sulfur 0.3%; 121=RDP 60%, NFC 40%, Sulfur 0%; 122=RDP 60%, NFC 40%, Sulfur 0.15%; 123=RDP 60%, NFC 40%, Sulfur 0.3%; 211=RDP 65%, NFC 35%, Sulfur 0%; 212=RDP 65%, NFC 35%, Sulfur 0.15%; 213=RDP 65%, NFC 35%, Sulfur 0.3%; 221=RDP 65%, NFC 40%, Sulfur 0%; 222=RDP 65%, NFC 40%, Sulfur 0.15%; 223=RDP 65%, NFC 40%, Sulfur 0.3%, IVDMD=*In vitro* of dry matter digestibility, IVOMD=*In vitro* of organic matter digestibility, IVADFD=*In vitro* of Acid detergent fiber digestibility, IVNDFD=*In vitro* of neutral detergent fiber digestibility, RDP=Rumen degradable protein, RUP=Rumen undegradable protein, NFC=Non-fiber carbohydrate Superscript ^a,b,c,d,e,f^ in different parameter and observation time means significantly different (p > 0.05)

### Characteristics of rumen fermentation

**Figure-1:**
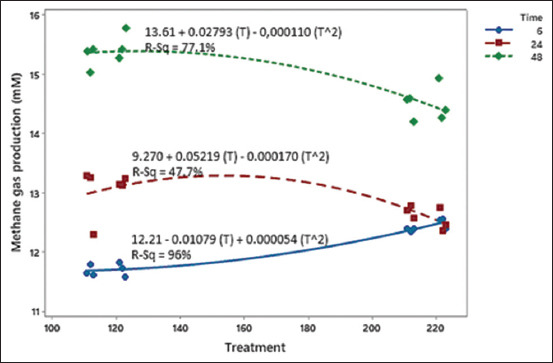
Correlation between RDP:NFC:Sulfur ration and methane gas production of experimental diets. NFC=Non-fiber carbohydrate, RDP=Rumen degradable protein.

The synchronization treatment between RDP: NFC:Sulfur did not influence the pH value, total and partial VFA, and methane gas production (p > 0.05). Several factors, including total VFA, iso-butyrate, acetate, and valerate, exhibited noteworthy significance (p < 0.05) due to variations in RDP content. NH_3_ and MPS levels were noticeably (p < 0.05) influenced by the RDP: NFC:Sulfur synchronization. NH_3_ levels ranged from 13.61 mg/100 mL to 25.63 mg/100 mL of rumen fluid. MPS concentrations ranged from 197.43 to 249.09 mg/100 mL. The total VFA concentration ranged from 113.43 to 118.77 mM. The pH value ranges from 6.59 to 6.90. Tables-3 and 4 present variations in the characteristic values of rumen fermentation. In Figure-1, a relationship is evident between the experimental diet and the extent of ruminal methane gas production, where the incubation periods of 6, 24, and 48 h showed a downward trend in methane gas emissions. The estimated ruminal methane production ranged from 11.58 to 15.77 mM.

**Table-3:** Total-VFA and Individual VFA (mM) of experimental diets.

Time	Total-VFA											

	111	112	113	121	122	123	211	212	213	221	222	223
6 h	101.68	101.7	100.41	101.28	101.26	100.88	102.12	102.36	102.83	102.93	102.27	102.21
24 h	107.59	108.16	105.96	108.55	108.3	107.85	106.81	105.9	106.34	106.21	105.95	106.2
48 h	117.31	116.88	116.53	116.42	116.52	118.77	114.82	114.73	114.77	114.41	113.43	113.72

**Time**	**Acetate**											

	**111**	**112**	**113**	**121**	**122**	**123**	**211**	**212**	**213**	**221**	**222**	**223**

6 h	23.03	23.19	22.52	23.11	23.1	22.73	24.24	24.32	24.56	24.69	24.45	24.14
24 h	27.07	27.35	25.35	27.35	27.31	27.21	26.07	25.83	25.72	25.72	25.26	25.49
48 h	31.8	31.16	31.56	31.28	31.48	32.37	29.22	29.62	28.36	29.76	28.51	28.84

**Time**	**Propionate**											

	**111**	**112**	**113**	**121**	**122**	**123**	**211**	**212**	**213**	**221**	**222**	**223**

6 h	17.58	17.33	16.88	17.13	17.35	17.27	17.07	17.16	17.38	17.23	16.8	16.83
24 h	18.87	19.31	19.28	19.53	19.51	18.95	19.06	18.39	18.95	18.23	18.85	18.85
48 h	20.92	21.19	20.43	20.5	20.38	21.35	20.5	20.68	20.91	19.95	20.23	20.23

**Time**	**Butyrate**											

	**111**	**112**	**113**	**121**	**122**	**123**	**211**	**212**	**213**	**221**	**222**	**223**

6 h	15.27	15.3	15.29	15.3	15.24	15.26	15.23	15.25	15.26	15.41	15.41	15.39
24 h	15.71^a^	15.63^ab^	15.46^bc^	15.50^bc^	15.47^bc^	15.50^bc^	15.53^bc^	15.52^bc^	15.51^bc^	15.45^bc^	15.42^c^	15.41^c^
48 h	17.07	17.09	17.06	17.08	17.11	17.69	17.62	17.38	17.94	17.53	17.46	17.43

**Time**	**Iso-butyrate**											

	**111**	**112**	**113**	**121**	**122**	**123**	**211**	**212**	**213**	**221**	**222**	**223**

6 h	15.26	15.28	15.26	15.26	15.20	15.23	15.20	15.23	15.22	15.22	15.22	15.23
24 h	15.47	15.39	15.34	15.40	15.36	15.40	15.40	15.40	15.40	15.46	15.41	15.45
48 h	15.81	15.78	15.79	15.84	15.84	15.76	15.78	15.66	15.82	15.72	15.77	15.76

**Time**	**Iso-valerate**											

	**111**	**112**	**113**	**121**	**122**	**123**	**211**	**212**	**213**	**221**	**222**	**223**

6 h	15.30	15.35	15.26	15.28	15.21	15.21	15.22	15.22	15.23	15.22	15.22	15.23
24 h	15.27f	15.27f	15.33^e^f	15.46^cd^	15.41^de^	15.47^cd^	15.43^de^	15.46^cd^	15.44^cde^	15.77^a^	15.56^bc^	15.59^b^
48 h	15.97^ab^	15.95^ab^	15.96^ab^	15.98^ab^	15.97^ab^	15.92^ab^	15.99^ab^	15.81^b^	16.02^a^	15.84^ab^	15.85^ab^	15.85^ab^

**Time**	**Valerate**											

	**111**	**112**	**113**	**121**	**122**	**123**	**211**	**212**	**213**	**221**	**222**	**223**

6 h	15.24^ab^	15.25^ab^	15.20^ab^	15.20^ab^	15.16^b^	15.18^ab^	15.16^b^	15.18^ab^	15.18^ab^	15.16^b^	15.17^ab^	15.39^a^
24 h	15.20^c^	15.21^c^	15.20^c^	15.31^bc^	15.24^bc^	15.32^bc^	15.32^abc^	15.30^abc^	15.32^abc^	15.58^a^	15.45^a^	15.41^a^
48 h	15.74	15.71	15.73	15.74	15.74	15.68	15.71	15.58	15.72	15.61	15.61	15.61

111=RDP 60%, NFC 35%, Sulfur 0%; 112=RDP 60%, NFC 35%, Sulfur 0.15%; 113=RDP 60%, NFC 35%, Sulfur 0.3%; 121=RDP 60%, NFC 40%, Sulfur 0%; 122=RDP 60%, NFC 40%, Sulfur 0.15%; 123=RDP 60%, NFC 40%, Sulfur 0.3%; 211=RDP 65%, NFC 35%, Sulfur 0%; 212=RDP 65%, NFC 35%, Sulfur 0.15%; 213=RDP 65%, NFC 35%, Sulfur 0.3%; 221=RDP 65%, NFC 40%, Sulfur 0%; 222=RDP 65%, NFC 40%, Sulfur 0.15%; 223=RDP 65%, NFC 40%, Sulfur 0.3%, VFA=Volatile fatty acids, NFC=Non-fiber carbohydrate, RDP=Rumen degradable protein, Superscript ^a,b,c,d,e,f^in different parameter and observation time means significantly different (p > 0.05)

**Table-4:** pH, NH3 and MPS of experimental diets.

Time	pH											

	111	112	113	121	122	123	211	212	213	221	222	223
6 h	6.94	6.91	6.88	6.91	6.81	6.98	6.99	6.98	6.96	6.92	6.96	6.97
24 h	6.97	6.99	6.82	6.87	6.87	6.63	6.63	6.67	6.81	6.92	6.85	6.84
48 h	6.69	6.68	6.89	6.59	6.65	6.78	6.73	6.88	6.82	6.77	6.90	6.85

**Time**	**NH_3_ (mg/100mL)**											

	**111**	**112**	**113**	**121**	**122**	**123**	**211**	**212**	**213**	**221**	**222**	**223**

6 h	4.00^bc^	4.47^bc^	3.12^e^f	2.77f	3.83^de^	5.53^b^	5.38^b^	6.17^a^	5.59^ab^	5.53^b^	5.51^b^	5.81^ab^
24 h	8.08^b^	8.93^ab^	10.42^ab^	8.51^ab^	8.93^ab^	10.63^ab^	12.12^a^	10.84^ab^	9.99^ab^	9.35^ab^	9.72^ab^	9.14^ab^
48 h	17.43^bc^	19.34^b^	16.13^bc^	13.61^c^	17.64^bc^	18.28^bc^	20.16^ab^	19.95^b^	22.32^ab^	21.75^ab^	21.79^ab^	25.63^a^

**Time**	**MPS (mg/100mL)**											

	**111**	**112**	**113**	**121**	**122**	**123**	**211**	**212**	**213**	**221**	**222**	**223**

6 h	93.18^e^	95.00^e^	122.19^c^	92.28^e^	108.59^d^	87.74^e^	177.48^ab^	182.92^a^	178.39^ab^	171.14^b^	178.39^ab^	171.14^b^
24 h	191.99^d^	191.99^d^	191.99^d^	183.83^e^	194.71^d^	184.74^e^	238.22^b^	234.59^c^	250.00^a^	234.59^b^	227.34^c^	220.99^c^
48 h	200.14^d^	211.02^c^	200.61^d^	197.43^d^	198.33^d^	199.24^d^	243.65^ab^	243.65^ab^	249.09^a^	240.03^ab^	247.28^a^	236.37^b^

111=RDP 60%, NFC 35%, Sulfur 0%; 112=RDP 60%, NFC 35%, Sulfur 0.15%; 113=RDP 60%, NFC 35%, Sulfur 0.3%; 121=RDP 60%, NFC 40%, Sulfur 0%; 122=RDP 60%, NFC 40%, Sulfur 0.15%; 123=RDP 60%, NFC 40%, Sulfur 0.3%; 211=RDP 65%, NFC 35%, Sulfur 0%; 212=RDP 65%, NFC 35%, Sulfur 0.15%; 213=RDP 65%, NFC 35%, Sulfur 0.3%; 221=RDP 65%, NFC 40%, Sulfur 0%; 222=RDP 65%, NFC 40%, Sulfur 0.15%; 223=RDP 65%, NFC 40%, Sulfur 0.3%, MPS=Microbial protein synthesis, NFC=Non-fiber carbohydrate, RDP=Rumen degradable protein, Superscript ^a,b,c,d,e,f^ in different parameter and observation time means significantly different (p > 0.05)

## Discussion

### Digestibility of dry matter and organic matter

As the incubation time for feed fermentation increases, the digestibility of both IVDMD and IVOMD becomes higher. The optimal incubation time is 48 h. The RDP:NFC 65 and 40 treatment and 1.5% sulfur supplementation resulted in the highest digestibility As a mineral source, sulfur triggers an increase in the rumen microbial population, thereby optimizing the digestibility [12]. Supplementation with 0.15% sulfur minerals in the diet at an RDP: NFC ratio of 65:40 produced the highest digestibility. This is consistent with the findings of Pazla *et al*. [12], in which the highest digestibility was observed with 0.17% sulfur mineral supplements. Putri *et al*. [13] reported that feed containing high crude fiber (CF) will thicken plant cell walls to reduce digestibility. On the other hand, feed ingredients are easier to digest if they contain a low amount of CF because the cell walls of the ingredients are thin, so rumen microbes easily degrade them.

According to Antonius *et al*. [14], an elevated breakdown of organic matter in the diet corresponds to increased nutrient sufficiency for livestock. The digestibility pattern of organic matter is similar to that of DM digestibility. The reason is that most of the dry material is organic. High digestibility increases livestock productivity because nutrients are optimally used [15]. In addition, the nutrient content of the feed plays an important role to play in influencing increased nutrient digestibility. Lv *et al*. [16] and Hao *et al*. [17] have similarly documented that a well-balanced nutrient supply leads to heightened microbial growth and improved nutrient digestibility, leading to enhanced microbial growth.

### Degradable and undegradable proteins

To meet the protein requirements of ruminant animals, several factors must be considered. These factors include the provision of N to support microbial growth, resistance to degradation, the ability to supply high bypass protein, and a high biological value [18]. Ruminants rely on degradable proteins and bypass proteins for productivity. In addition, protein solubility in the feed affects protein degradation within the rumen. Feed with a high level of solubility will undergo rapid degradation in the rumen. However, feed with high protein solubility but disulfide bonds tends to undergo slow degradation and may even penetrate the post-rumen. In this study, rumen protein digestibility (RDP) increased with increasing RDP and NFC content, indicating that the synchronization of RDP and NFC positively affected rumen microbial activity. In line with the previous research by Putri *et al*. [13], increasing RDP positively influences rumen microbes in providing N for protein synthesis. These results are also in line with other research reports that a diet with an RDP: NFC ratio of 60:35 produces the most optimal digestibility [19]. High rumen microbial activity increases nutrient digestibility due to increased RDP [20]. Increasing RUP content can reduce rumen microbial activity due to the low N supply for rumen MPS, thereby reducing digestibility [13, 21, 22].

Proteins degraded in the rumen (RDP) are converted into NH_3_, which is later used for MPS. The walls of the rumen absorb ammonia, which is quickly released, and the microbes use some of it. The activity of rumen microbes in synthesizing their body protein depends on the pH value, rumen microbes, organic matter, and DM of the feed, feed sources of easily fermentable energy, N compounds, balance of feed sources of energy and protein, ratio of forage and concentrate, and feed rate in the rumen [23]. Crude protein is a nutrient in feed that determines the process of MPS because protein degradation shows that N compounds for rumen microbes can be available, but the N concentration must be sufficient and the energy source does not come from protein [24]. When N availability is in harmony with the energy in the rumen, it promotes an increase in MPS, resulting in increased microbial activity and feed digestion ability. This resulted in augmented *in vitro* MPS in diets with elevated RDP levels [25].

### Fiber fraction digestibility (Acid detergent fiber [ADF] and Neutral detergent fiber [NDF])

The highest digestibility of ADF and NDF was observed in the 65% RDP, 40% NFC, and 1.5% sulfur supplementation treatments. A smaller fiber content may increase digestibility because rumen microbes will digest it more easily. According to Wahyono *et al*. [26], a reduced fiber fraction component makes cellulose and hemicellulose digestion more accessible to microorganisms and increases digestibility. In this study, the fiber fraction content (ADF and NDF) decreased with increasing RDP and NFC content. The highest ADF and NDF digestibility in the RDP: NFC:Sulfur 65:40:1.5 treatment resulted in the lowest ADF and NDF content among all treatments (Table-1). In addition, the level of lignin (LIG) in the feed is one of the factors that affect digestibility. LIG binds to the fiber fraction, making it difficult for rumen microbes to break down [27]. Therefore, it is important to balance RDP and NFC in high-fiber diets to optimize rumen microbial activity. Hao *et al*. [17] emphasized that a well-balanced nutrient supply provides ample substrate for rumen microbes, which promotes their growth and improves nutrient digestibility.

### Characteristics of rumen fermentation

In this study, the rumen pH value was found to fall within the standard range, which typically ranges from 5 to 7 [27]. The pH value of the rumen, a factor that determines the rumen fermentation process [19], describes the good or bad condition of the rumen. In addition to its nutritional content, a normal pH value in accordance with rumen conditions will optimize the performance of microbes in digesting feed. This is because the pH of the rumen is suitable so that microbes are able to increase their activity in digesting the feed. Our findings suggest that environmental factors do not affect rumen pH. Rumen microbes thrive in a pH range of 6.0–7.0 and produce ammonia (NH_3_) and VFAs as byproducts of fermentation. It should be noted that this pH range does not influence the activity or composition of the rumen microbes [28]. In addition, it has cellulolytic microbial activity [29].

Ammonia (NH3) is a very important fermentation product and shows how much feed protein is degraded by rumen microbes [30]. In addition, protein quality in ruminant diet can be determined by measuring NH_3_ concentration [31]. The NH_3_ product in the rumen is used by rumen microbes for body synthesis. According to McDonald *et al*. [32], the NH_3_ concentration required for the growth of rumen microbes ranges between 85 and 300 mg/L. Leng [33] reported that the NH_3_ concentration in the rumen fluid varies between 1 and 34 mg/100 mL. The concentration of NH_3_ in this study was within the normal range (Table-4). Rumen NH_3_ products from protein degradation are absorbed in the rumen walls and some will be recycled again (N recycling); therefore, it is important in the metabolic processes of ruminant animals [34].

Ruminants are animals whose main energy source is rumen fermentation products, namely, VFAs. There were no differences in the total VFA concentrations between the treatments. The VFA values obtained in this study are consistent with those reported by Savari *et al*. [35] and Rosmalia *et al*. [19], who reported that RDP and NFC levels do not affect total VFA concentrations. High or low concentrations of VFA are influenced by several factors such as the speed at which the feed is fermented, the amount of substrate that is digested, the rate at which the feed is consumed, and the amount of VFA absorbed [36]. However, in this study, the synchronization of RDP: NFC:Sulfur did not affect the total VFA concentration. This result arises from the equilibrium between RDP and NFC release, which facilitates the optimization of protein synthesis within rumen microbes and the generation of elevated levels of fermentation products. VFA production occurs in the rumen, and these VFAs are then taken up by epithelial cells lining the rumen wall. Both VFA production and the equilibrium of absorption within the rumen wall influence the absorption process [37]. The type of feed, depolymerization speed and type of rumen microbes available in the rumen influence VFA production [19]. Acetate contains the highest proportion of all types of VFA. Glucose, the primary source of energy in ruminants, stems primarily from VFA propionate, which serves as a substrate for gluconeogenesis. In addition, acetate and butyrate are precursors of long-chain fatty acid formation [38]. Notably, the individual proportions of VFA remained unaffected by the synchronization of RDP, NFC, and sulfur supplementation in this study. However, the previous study has indicated that an increase in NFC leads to a heightened proportion of propionate [39]. It is assumed that this is due to the different NFC sources. However, further studies by Broderick *et al*. [40] and Paula *et al*. [41] underscored that dietary NFC has a limited impact on the rumen fermentation process, particularly in relation to propionate production. In addition, the molar concentration of n-butyrate increased with increasing NFC levels. Butyrate stimulates growth and expansion in rumen papillae [42]. Metabolic processes that entail the oxidative removal of amino groups and carboxyl groups from branched-chain amino acids (isoleucine, valine, and leucine) result in the formation of branched-chain VFAs, such as iso-valerate, iso-butyrate, and valerate. In addition, carbohydrates and carboxylic acids produce n-valerate [43]. Branched-chain amino acids (leucine, isoleucine, proline, and valine) are synthesized from branched-chain VFAs (such as iso-butyrate, iso-valerate, and n-valerate) produced by cellulolytic bacteria [43]. The balance between RDP and NFC in the diet significantly affected the amount of iso-valerate in the rumen fluid. These results differ from those of previous studies, which indicated that balancing RDP and NFC in the feed did not significantly affect iso-butyrate, n-valerate, and iso-valerate content [39].

### Microbial protein synthesis

Rumen microbial growth can be observed through MPS. The greater the number of microbes in the rumen, the more protein they produce [44]. The synthesis of microbial proteins results from the harmonious coordination of energy sources in the feed with proteins that are readily broken down in the rumen [16]. Ruminants obtain most of their protein from micro-organisms in their rumen. Ruminant animals require 80%–90% of their amino acids from microbial proteins [45]. Enhancing MPS requires an increase in the dietary RDP. Conversely, if there is more RUP in the diet, microbes will produce less protein, and the animal will be able to digest less of its food. This study shows that increasing the feed RDP and NFC can provide sufficient N from NH_3_ and carbon (C) atoms from VFA. High NFC in the diet can increase MPS because it provides carbohydrates and energy that can be quickly fermented [46]. Proteins and carbohydrates are the primary nutrients required for MPS. A balanced supply of N and C can be determined using the synchronization index formula [47], which calculates the amount of organic matter and N degraded per hour. MPS can also be enhanced by increasing the synchronization index [48]. High MPS levels are also influenced by several mineral and vitamin factors [23]. In this study, sulfur supplementation was able to increase MPS, so the MPS concentration was higher (Table-4) compared with prior research by Putri *et al*. [13] and Rosmalia *et al*. [19]. Sulfur supplementation increases the efficiency of MPS [49].

### Methane gas production

Rapid gas production at 24–48 h intervals can be attributed to the exponential growth phase of rumen microbes, which have adapted to their new environment and access to abundant substrates. These data are in accordance with research by Karabulut *et al*. [50], where gas production data almost doubled when the observation time interval was 12 and 24 h. In addition to producing VFA, the fermentation of feed in the rumen also produces gases such as CO_2_, CH_4_, NO_2_, and NO. These gases originate from the fermentation of organic substances in the feed consumed by animals. The quantity of gas produced depends on the nutritional content of the feed. Increased carbohydrate content in the feed leads to increased gas production in the rumen from feed fermentation. Increased activity of cellulolytic bacteria increases acetate production, leading to an increase in the acetate-to-propionate ratio [34]. Methane gas production originating from rumen fermentation positively correlates with the acetate and propionate ratio and is also an indicator of CH_4_ production [51]. Acetate and butyrate are VFAs that produce hydrogen (H) during its formation process, and propionate uses H atoms during its formation process. Methanogenic bacteria use the H atom to synthesize CH_4_, so increasing acetate and butyrate will increase methane production [52].

## Conclusion

Effective enhancement of rumen fermentation and MPS is achieved by supplementing rumens with a dietary RDP to NFC ratio. The research findings conclude that the interaction between RDP, NFC, and sulfur significantly enhances digestibility and reduces methane gas production. Feed digestibility was increased in the 65% RDP treatment with 40% NFC and 0.15% sulfur, along with a decrease in methane gas production.

## Authors’ Contributions

MZ and RP: Designed the study and drafted and reviewed the manuscript. EMP: Supervised field and laboratory works. UA, UHT, JAS, and MM: Performed field and laboratory works and tabulated data. YY, POS, and BB: Collected and prepared samples and performed data analysis. All authors have read, reviewed, and approved the final manuscript.
